# Three New Fungi from China: A Potentially Psychoactive *Psilocybe* and Two *Deconica* Species

**DOI:** 10.3390/jof11120887

**Published:** 2025-12-16

**Authors:** Hai-Ying Feng, Jia-Xin Li, Wen-Qiang Yang, Bin Cao, Rui-Lin Zhao

**Affiliations:** 1 State Key Laboratory of Microbial Diversity and Innovative Utilization, Chinese Academy of Sciences, Beijing 100101, China; fhy6244@126.com (H.-Y.F.); lijiaxin18@mails.ucas.ac.cn (J.-X.L.); yangwq_107@163.com (W.-Q.Y.); caob@im.ac.cn (B.C.); 2 College of Life Sciences, University of Chinese Academy of Sciences, Beijing 100049, China; 3 Xizang Key Laboratory of Plateau Fungi, Institute of Plateau Biology of Xizang Autonomous Region, Lhasa 850000, China; 4 Department of Biology, Faculty of Science, Chiang Mai University, Chiang Mai 50200, Thailand

**Keywords:** *Psilocybe*, *Deconica*, multigene, phylogeny, taxonomy

## Abstract

The saprotrophic genera *Deconica* and *Psilocybe* exhibit broad geographic distributions across temperate and subtropical biomes globally. Despite China’s rich fungal diversity, taxonomic studies of these genera remain limited, with few species previously documented. In this study, 64 specimens of *Deconica* and *Psilocybe* from China were examined using an integrative taxonomic approach combining detailed morphological characterization with multilocus phylogenetic analyses (ITS, nrLSU, *rpb2*, and *tef1-α*). We identified six known *Psilocybe* species, three known *Deconica* species, and three species new to science, i.e., *Deconica lignicola*, *D*. *shannanensis*, and *Psilocybe striata*. In addition, at least six not yet described taxa were identified as members of the two genera. *Psilocybe striata* is distinguished by a white pileus covered with distinctive striate veils and exhibits a bluing reaction, which was discovered in Chongqing, representing the expansion of the known geographic range of *Psilocybe* in China. *Deconica lignicola* is characterized by pleurocystidia of the chrysocystidia type and narrowly lageniform cheilocystidia with short necks, and it is a typical lignicolous fungus, which grows on decaying wood. *Deconica shannanensis* produces small, reddish-brown to dark brown basidiomata, with abundant narrowly lageniform to lageniform cheilocystidia and does not have pleurocystidia; it is a coprophilous species inhabiting high-altitude meadows. These findings not only enrich the recorded species diversity of *Deconica* and *Psilocybe* in China but also deepen the understanding of their ecological adaptations and geographical distribution.

## 1. Introduction

The *Psilocybe* (Fr.) P. Kumm., typified by *P*. *semilanceata* (L.) P. Kumm., belongs to Hymenogastraceae, Agaricales, and is notable for its psychoactive properties [1,2]. All currently known species of *Psilocybe* are saprotrophic, exhibiting a cosmopolitan distribution across tropical, subtropical, and temperate biomes [3,4]. They preferentially colonize nutrient-rich substrates, such as decaying wood, leaf litter, and herbivore dung, playing a crucial role in ecosystem nutrient cycling through the enzymatic degradation of recalcitrant biopolymers like cellulose and lignin [5,6,7,8]. Morphologically, the key diagnostic feature of *Psilocybe* species is the rapid bluing reaction of the basidiomata upon mechanical injury, caused by the oxidation of psilocybin [9,10,11]. Microscopically, their basidiospores are smooth-walled, with a distinct germ pore, and clamp connections are commonly present at septa [12,13]. Species within *Psilocybe* biosynthesize neuroactive indole alkaloids, notably psilocybin and psilocin, rendering them significant subjects in ethnomycology and neuropharmacology [6,7,14,15,16]. Some species engage in dynamic interactions with soil microbial consortia. For instance, the mycelial networks of *P*. *cubensis* can selectively enrich specific bacterial taxa (e.g., *Ochrobactrum* and *Stenotrophomonas*) via exudate-mediated metabolic cross-talk, thereby promoting phytohormone biosynthesis and facilitating plant growth [17].

Although phylogenetically distinct, *Deconica* (W.G. Sm.) P. Karst. closely resembles *Psilocybe* in basidiome structure and spore morphology [6,18]. However, *Deconica* species are consistently distinguished by the absence of psychoactive indole alkaloids and the consequent lack of a bluing reaction upon injury. Additionally, some *Deconica* species possess chrysocystidia (cystidia with refractive golden-yellow contents), in contrast to their absence in *Psilocybe*. *Deconica* exhibits a broad distribution across temperate and subtropical regions, occupying diverse niches from primary forests to human-disturbed habitats, commonly fruiting on decaying wood, mosses, dung, and soil [18,19,20].

The taxonomic history of *Psilocybe* and *Deconica* has been a persistent point of contention. Historically circumscribed based on morphological characteristics, they were previously classified under *Psilocybe* (Fr.) P. Kumm. based on traditional taxonomic frameworks [21]. However, molecular phylogenetic studies later revealed this broad concept to be polyphyletic. This evidence necessitated a revision, leading to the use of the genus *Deconica* by Redhead et al. (2007) to accommodate the non-bluing, non-psychoactive taxa, while retaining the psychoactive, bluing species within *Psilocybe* [2]. This reclassification was formalized by Noordeloos (2009) and subsequently corroborated by multilocus phylogenetic analyses [6,22], resolving long-standing historical misidentifications and taxonomic ambiguities.

Despite global advances in the taxonomy and phylogeny of *Psilocybe* and *Deconica*, research on the two genera in China remains limited. Knowledge concerning their species diversity, distribution patterns, and phylogenetic relationships within the region is particularly scarce. The *Flora Fungorum Sinicorum* (2014) listed only 11 *Psilocybe* species and provides a systematic description of these taxa [12]. Although these species were clearly distinguished based on morphological differences, the study was limited primarily to species descriptions and lacked extensive molecular or ecological analyses. A subsequent update by Tolgor et al. (2024) expanded the list to 15 species and supplemented it with information on their ecological roles and distributions, but molecular data are still limited [13]. In the past five years, only two new species, *P*. *zhushanensis* (R.L. Zhao and B. Cao) and *P*. *ningshanensis* (X.L. He, W.Y. Huo, L.G. Zhang, Y. Liu, and J.Z. Li), have been described in China [23,24]. For *Deconica*, the genus is estimated to comprise approximately 133 taxa [3]. Although extensive phylogenetic studies have been conducted worldwide on *Deconica*, such as those by Ramírez-Cruz et al. (2013) and Silva et al. (2013) that provided additional molecular data and phylogenetic insights, taxonomic research on this genus in China has remained relatively limited [6,25]. This long-standing gap was not addressed until the recent work of Yan et al. (2024) and Shen et al. (2024), who described a total of five new species, marking a pivotal step in exploring *Deconica* diversity in China [18,26].

Research on these genera has mainly focused on taxonomy and distribution, with growing interest in their psychoactive compounds. A revised checklist of poisonous mushrooms in China lists 24 *Psilocybe* species, 15 of which contain psychoactive compounds like psilocybin, responsible for neuropsychiatric syndrome poisoning [27,28]. *Psilocybe* is the primary group of hallucinogenic mushrooms, with approximately 144 species documented [3]. Psilocybin has shown significant clinical efficacy in treating depression and post-traumatic stress disorder [29,30].

The aim of this study was to conduct an integrative taxonomic study combining detailed morphological characterization with multilocus phylogenetic analyses. Targeted loci included the Internal Transcribed Spacer (ITS), the nuclear large ribosomal subunit (nrLSU), the second largest subunit of RNA polymerase II (*rpb2*), and the translation elongation factor 1-α (*tef1-α*). Based on 64 specimens of the genera *Deconica* and *Psilocybe* collected from China, we identified nine known species and described three new species: *Deconica lignicola*, *D*. *shannanensis*, and *Psilocybe striata*. The generic names *Psilocybe* and *Deconica* are abbreviated as *P*. and *D*., respectively.

## 2. Materials and Methods

### 2.1. Specimen Collection and Morphological Study

Fresh basidiomata of *Psilocybe* and *Deconica* were collected from natural habitats. Macro-morphological characteristics, including color changes upon handling or bruising, odor, and substrate, were documented in situ with photographic records. Specimens were carefully wrapped in aluminum foil to prevent damage or mixing during transport. Upon return to the lab, specimens were desiccated to constant mass using a food drier at 50 °C, subsequently stored in sealed plastic bags and deposited in the Mycological Herbarium of the Chinese Academy of Sciences, Beijing (HMAS).

Micro-morphological analyses followed Largent et al. (1986) [31]. Dried specimens of mature basidiomata were rehydrated in 5% KOH, stained with 1% Congo red, and observed for microstructures such as basidiospores, basidia, cheilocystidia, and pleurocystidia. The abbreviation [n/m/p] denotes measurements of n basidiospores derived from m basidiomata across p distinct specimens. Basidiospore dimensions are expressed as (a) b–c (d), where the range b–c encompasses 90% of measured values, with a and d representing minimum and maximum extremes, respectively. In addition, x denotes the mean aspect ratio ± standard deviation (SD). Q is the aspect ratio of basidiospores observed from the side, and Qm denotes the mean of all basidiospores ± is the standard deviation of the sample [31,32,33].

### 2.2. DNA Extraction, PCR Amplification, and Sequencing

Genomic DNA was extracted from dried specimens using a Broad-spectrum plant Rapid Genomic DNA Kit (Biomed, Tokyo, Japan) according to the manufacturer’s protocol. The extracted DNA was used as a template for PCR amplification of four gene regions (ITS, nrLSU, *rpb2,* and *tef1-α*). Primers ITS1-F and ITS4 were used for sequencing internal transcribed spacer (ITS) [34,35], while LROR and LR5 were used for large ribosomal subunit (nrLSU) [36]. The *rpb2* gene was sequenced with primers brpb2-6F/brpb2-7.1R, and the *tef1-α* gene was targeted with EF1-983F/EF1-1567R [37,38]. The PCR programs followed Li et al. (2025) [33]. The PCR products were purified and sequenced by Beijing Tianyi Huiyuan Biotechnology Co., Ltd. (Beijing, China).

### 2.3. Molecular Phylogenetic Study

Sequence checking and assembly were performed using Geneious Prime v2025.0.3 [39]. Raw sequences were aligned using AliView v1.28 with the MAFFT algorithm [40], followed by manual curation to trim ambiguous positions and gaps. These sequences were then concatenated in PhyloSuite v1.2.3 [41], with missing data and ambiguous regions coded as “N”. Phylogenetic analysis was conducted using both Maximum Likelihood (ML) and Bayesian Inference (BI) methods. The ML tree was constructed by RAxML v7.2.6 using the GTR+G+I site substitution model, which includes a General Time Reversible (GTR) substitution matrix, Gamma-distributed rate heterogeneity among sites, and a proportion of invariant sites, and branch support was evaluated with 1000 bootstrap (BS) replicates [42]. BI analyses were performed with MrBayes v3.2.7a, utilizing a general time reversible (GTR) model of DNA substitution and a gamma distribution rate variation across sites [33,43,44]. Four chains were processed with 10 million generations for each gene. All phylograms were visualized with iTOL v5 and graphically refined in Adobe Illustrator 2024 (Adobe Inc., San Jose, CA, USA) [45]. All newly generated nucleotide sequences from this study (ITS, nrLSU, *rpb2*, and *tef1-α*) have been deposited in the GenBank database.

## 3. Results

### 3.1. Molecular Phylogenetic Analysis

**Figure 1:**
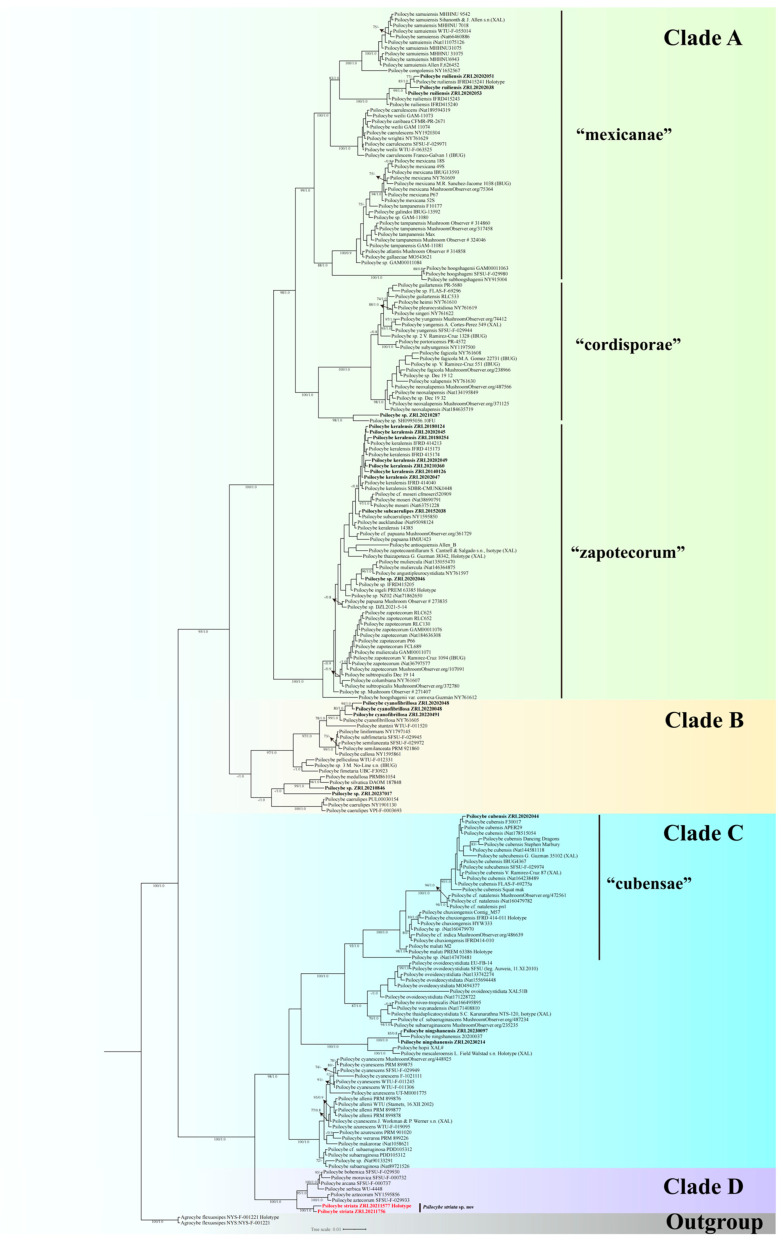
Bayesian Inference (BI) tree of *Psilocybe* inferred from ITS, nrLSU, *rpb2*, and *tef1-α* sequences. BS > 70% and PP > 0.80 are indicated on nodes. Examined specimens in this study are shown in boldface, and new species are highlighted in bold red.

**Figure 2:**
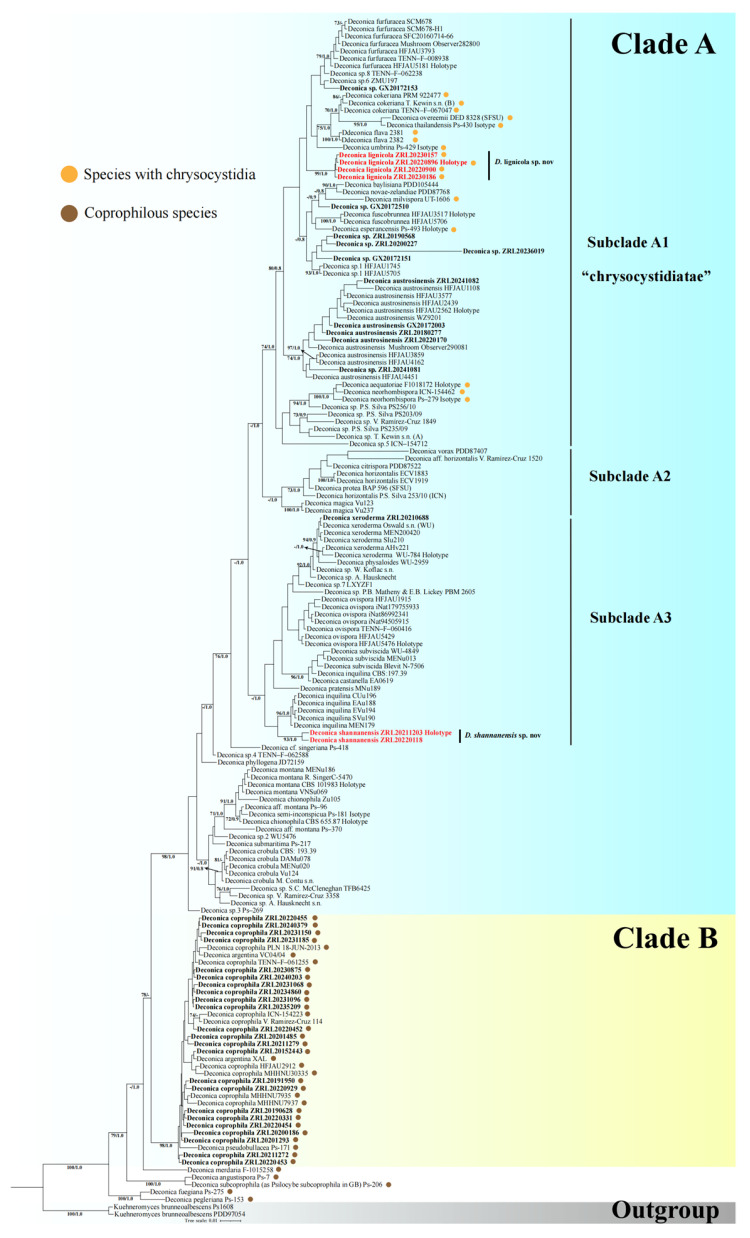
Maximum likelihood (ML) tree of *Deconica* inferred from ITS, nrLSU, *rpb2*, and *tef1-α* sequences. BS > 70% and PP > 0.80 are indicated on nodes. Examined specimens in this study are shown in boldface, new species are highlighted in bold red, and species with chrysocystidia are marked with orange dots, while coprophilous species are marked with brown dots.

Two separate combined datasets were used for multigene phylogenetic analysis of *Psilocybe* and *Deconica*. The first *Psilocybe* dataset comprised 364 sequences (191 ITS, 71 nrLSU, 49 *rpb2,* and 53 *tef1-α*) retrieved from previous studies [6,14,46,47,48,49,50], along with 89 newly generated sequences (23 ITS, 23 nrLSU, 20 *rpb2,* and 23 *tef1-α*), while *Agrocybe* was selected as the outgroup. The Maximum Likelihood (ML) and Bayesian Inference (BI) analyses exhibited similar topologies. The ML tree is presented in Figure 1. The second *Deconica* dataset, with *Kuehneromyces* selected as the outgroup, comprised 142 sequences (121 ITS and 21 nrLSU) obtained from previous studies [6,18,20,26], and 128 newly generated sequences (41 ITS, 33 nrLSU, 24 *rpb2,* and 30 *tef1-α*). The Maximum Likelihood (ML) and Bayesian Inference (BI) analyses produced congruent topologies. The ML tree is presented in Figure 2. All sequences of *Psilocybe* and their GenBank accession numbers are shown in Table S1, while those of *Deconica* are listed in Table S2; sequences newly generated in this study are shown in boldface.

In the phylogenetic tree (Figure 1), the results robustly support *Psilocybe* as a monophyletic clade with strong statistical support (BS = 100%; PP = 1.00), which is divided into four major clades (A–D). These four clades are resolved into two major sister lineages: one comprising Clades A and B, and the other comprising the sister Clades C and D. This topology is consistent with prior studies [6,14,48,49,50,51]. Clade A (BS = 100%; PP = 1.00) comprises subclades mexicanae, cordisporae, and zapotecorum. Clade B (PP = 1.0) exhibited conflicting positions between the Maximum Likelihood (ML) and Bayesian Inference (BI) analyses. In the BI tree, it formed a distinct, strongly supported cluster; in contrast, it failed to resolve as a monophyletic group in the ML tree (Figure S1). Clade C (BS = 98%; PP = 1.00) is characterized by a group of widely distributed species, including the well-known *P*. *cubensis*, *P*. *cyanescens,* and their close relatives. Clade D (BS = 100%; PP = 1.00) was clustered as an independent lineage comprising the *P*. *serbica* species complex alongside the newly described *P*. *striata*. This placement of the *P*. *serbica* complex into a distinct Clade D contrasts with Ramírez-Cruz et al. (2013) but is strongly supported by more recent studies [14,24,48]. In our analysis, the *P*. *cyanescens* group remains within Clade C, reinforcing the separation of these two lineages.

As shown in Figure 2 and Figure S2, *Deconica* is strongly supported as a monophyletic group (BS = 100%; PP = 1.0), consistent with previous studies [6,18,20,26]. Within *Deconica*, Clade A (BS = 98%; PP = 1.0) consists of three subclades (A1–A3). Subclade A1 (BS = 74%; PP = 1.0) corresponds to the “chrysocystidiatae” clade, whose members are predominantly characterized by the presence of chrysocystidia. Subclade A2 (PP = 1.0), which lacks strong support in the ML analysis, groups taxa exhibiting reduced or absent stipes and small basidiospores. Subclade A3 (PP = 1.0) comprises species that display small to moderately sized basidiomes, basidiospores with thin to slightly thickened walls, cheilocystidia, and no pleurocystidia. Similar to A2, this subclade lacks significant support in the ML tree but is strongly supported in the BI analysis; its monophyly is also corroborated by shared morphological traits. Outside Clade A, the phylogeny includes a group of coprophilous taxa. Among these, Clade B (BS = 98%; PP = 1.0) constitutes a well-supported phylogenetic complex comprising *D*. *coprophila*, *D*. *argentina*, and *D*. *pseudobullacea*. However, resolving the relationships within this complex and among the other coprophilous species requires integration of additional molecular data and morphological characterization.

Based on these well-supported phylogenetic placements and morphological evidence, one new species of *Psilocybe* and two of *Deconica* are proposed herein. All novel taxa form monophyletic groups with full statistical support (Figure 1 and Figure 2). Detailed morphological descriptions, photographs, and diagnostic comparisons are presented in the Section 3.2.

### 3.2. Taxonomy

**Figure 3:**
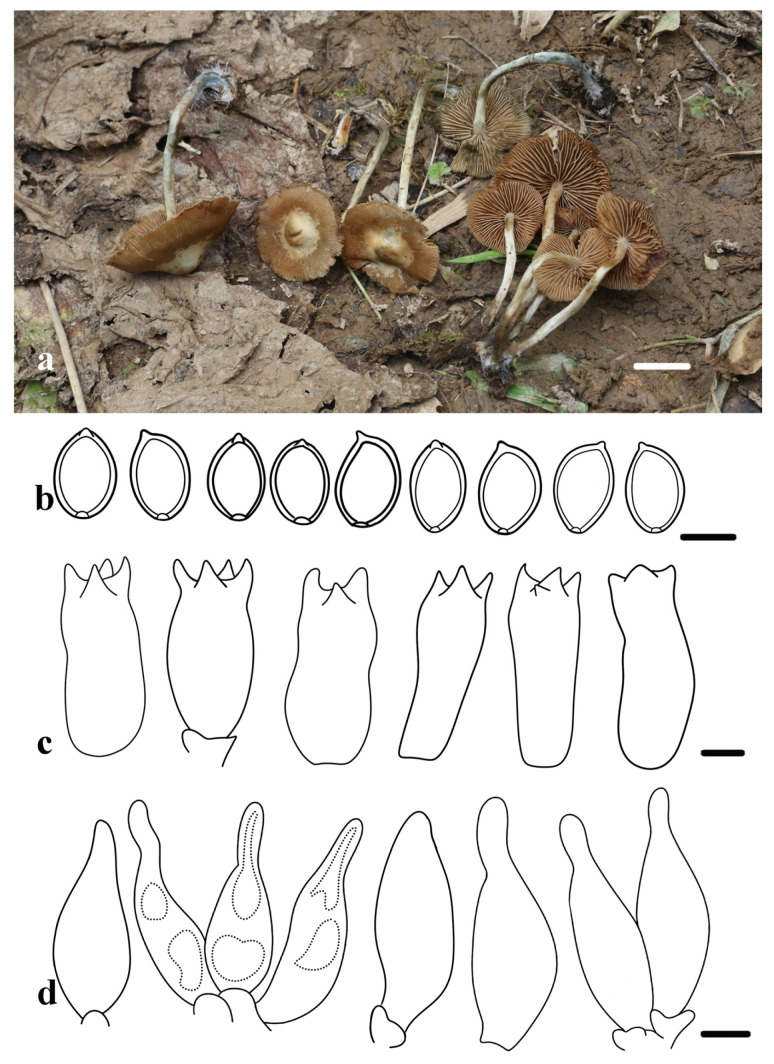
Morphology of *Psilocybe striata*. (**a**) Basidiomata (holotype, HMAS 282652). (**b**) Basidiospores. (**c**) Basidia. (**d**) Cheilocystidia. Scale bars: (**a**) = 1 cm, (**b**–**d**) = 5 µm.

***Psilocybe striata*** R.L. Zhao, H.Y. Feng, J.X. Li, and B. Cao, sp. nov. Figure 3.

Fungal Names: FN 572999

*Etymology*: *striata* (Latin) meaning “striped”, referring to the conspicuously striate pileus surface of the species.

*Type*: China, Chongqing, Kaiju County, Xuebaoshan National Nature Reserve, Xiaoyuan village, 31°35′17″ N, 108°41′18″ E, 1350 m a.s.l., 11 September 2021, X.Y. Zhu, *ZRL20211577* (holotype HMAS 282652), Genbank accession: PV940825 (ITS), PV940896 (LSU), PX394216 (*rpb2*), PX401805 (*tef1-α*).

*Diagnosis*: *Psilocybe striata* is characterized by a pileus with a whitish background overlaid with striate, detachable yellowish-brown veils, an irregular margin with overhanging veil remnants, and a stipe base with dense white rhizomorphs. Microscopically, it produces ellipsoid, slightly thick-walled basidiospores and conical or lageniform cheilocystidia. Basidiomata turn blue upon mechanical damage.

*Macroscopic description*: Pileus 16–33 mm, initially campanulate, becoming plano-convex to applanate with age, developing a beige broad central papilla or umbo; surface whitish with detachable yellowish-brown to sepia veils, radially striate from margin to papilla and with white spots; margin irregular, often with overhanging veils remnants, bluing when mechanical injury. Context thin, yellowish centrally. Lamellae adnexed to adnate, with numerous lamellulae of varying lengths, situated between lamellae; color congruent with pileus or slightly darker, edges undulate, sometimes with scattered hyaline crystalline deposits. Stipe 25–38 × 1.5–2.5 mm, central, cylindrical, base slightly swollen, surface beige with some brownish, and squamulose fibers at the apex, base with dense white rhizomorphs and a distinct bluing reaction upon injury. Odor not recorded.

*Microscopic description*: Basidiospores [60/3/2], (7.4) 8.3–9.5 (10.8) × (5.1) 5.6–6.4 (7.6) µm, X = 8.9 ± 0.6 × 6.0 ± 0.4 µm, Q = 1.4–1.6, Qm = 1.5 ± 0.1, ovoid or ellipsoid in frontal view, ellipsoid to subcitriform in lateral view, with germ pore, slightly thick-walled, smooth, yellowish brown or dark yellow in KOH. Basidia 21.1–241 × 9.2–10.3 µm, clavate, cylindrical to subcylindrical, thin-walled, hyaline, four-spored. Cheilocystidia 22.7–25.9 × 7.5–9.1 µm, abundant, few conical, often lageniform with a short neck, apex rounded or sometimes slightly capitate, occasionally with inclusions, thin-walled, hyaline. No pleurocystidia. Clamp connections are present at the base of basidia and cheilocystidia.

*Habitat and Habit*: Saprotrophic, scattered or gregarious on humus-containing plant debris or mosses in mixed coniferous-broadleaved forest. Currently known only from Chongqing, China.

*Additional specimens examined*: China, Chongqing, Wushi county, Yintiaolin National Nature Reserve, 31°23′52″ N, 109°41′19″ E, 2796.8 m a.s.l., 16 September 2021, X.Y. Zhu, *ZRL20211756* (HMAS 282653).

*Notes*: Phylogenetically, *P. striata* is deeply nested within Clade D, where it forms a distinct, strongly supported lineage. It is resolved as the sister group to a subclade containing *P*. *serbica*, *P*. *moravica*, *P*. *arcana*, *P*. *bohemica*, and *P*. *aztecorum* (Figure 1). Morphologically, *P*. *striata* shares several features with this group, but can be distinguished by a unique combination of characteristics. Macroscopically, all these species exhibit a bluing reaction. Additionally, *P*. *striata* shares the presence of conspicuous white rhizomorphs at the stipe base with *P*. *bohemica*, *P*. *moravica*, and *P*. *arcana*, which are absent in both *P*. *serbica* and *P*. *aztecorum* [52,53,54,55,56]. While all related species have ochraceous brown, thick-walled basidiospores with a germ pore, the basidiospores of *P*. *striata* are notably smaller (8.3–9.5 μm long) than those of its relatives, which typically exceed 10 μm in length [53,54,56]. Cystidial morphology provides the most definitive diagnostic characteristics. *Psilocybe striata* is characterized by the presence of conical or lageniform cheilocystidia (often with short necks) and does not have pleurocystidia, while *P*. *arcana*, which also lacks pleurocystidia, has lageniform cheilocystidia with more elongated necks [53]. In contrast, all other species in the complex possess pleurocystidia: *P*. *serbica* has lageniform pleurocystidia and cheilocystidia with elongated necks [53,56], *P*. *aztecorum* has pleurocystidia with long or rostrate necks [54,55,56], and both *P*. *moravica* and *P*. *bohemica* develop pleurocystidia, though in *P*. *bohemica*, these are often scarce [52,53,57]. Therefore, the unique combination of significantly smaller basidiospores, the presence of lageniform cheilocystidia, and no pleurocystidia provides a robust and clear morphological diagnosis for *P*. *striata* against all its closely related species.

**Figure 4:**
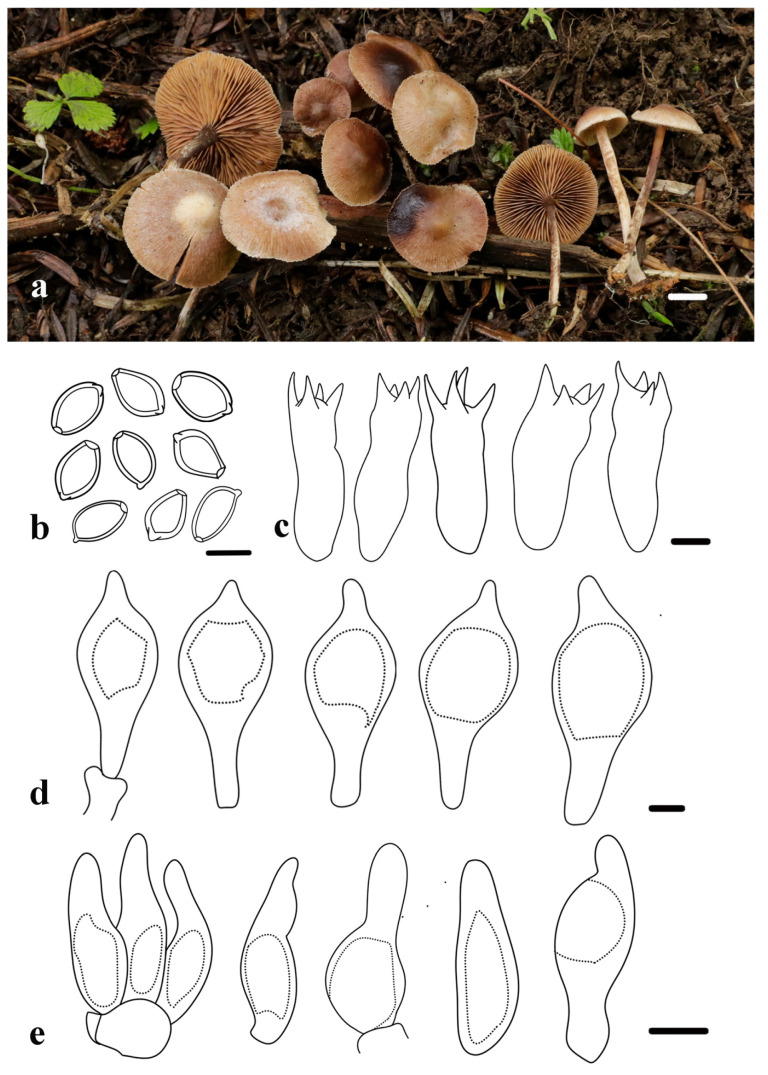
Morphology of *Deconica lignicola*. (**a**) Basidiomata (holotype, HMAS 282620). (**b**) Basidiospores. (**c**) Basidia. (**d**) Pleurocystidia. (**e**) Cheilocystidia. Scale bars: (**a**) = 1 cm, (**b**–**e**) = 5 µm.

***Deconica lignicola*** R.L. Zhao, H.Y. Feng, J.X. Li and B. Cao, sp. nov. Figure 4.

Fungal Names: FN 573000

*Etymology*: *lignicola* (Latin), meaning “wood-inhabiting”, referring mostly to its habit of growing on decaying wood.

*Type*: China, Hubei province, Shennongjia Forestry District, Shennong Peak Watchtower, 31°27′30″ N, 110°16′13″ E, 2886 m a.s.l., 26 Aug. 2022, X.Y. Zhu, *ZRL20220896* (holotype HMAS 282620), Genbank accession: PV940853 (ITS), PV940905 (LSU), PX376975 (*rpb2*), PX401783 (*tef1-α*).

*Diagnosis*: *Deconica lignicola* is characterized by its pileus with a pointed to broadly rounded beige papilla and radiating striations towards margins; Basidiospores ellipsoid or oblong-ellipsoid, slightly thick-walled with a germ pore; Pleurocystidia type chrysocystidia, fusiform, clavate to broadly lageniform; and narrowly lageniform cheilocystidia with a short neck.

*Macroscopic description*: Pileus 10–25 mm, juvenile pileus often campanulate, typically expanding to plano-convex at maturity, usually with a pointy to broadly rounded beige papilla; surface smooth, light brown to yellowish brown or sepia, sometimes with reddish brown tones; margin straight, crenate, with distinct striations from margin to papilla. Context thin, fleshy, and brown. Lamellae adnate, subdistant, with lamellulae and narrow interlamellar gaps, edges smooth without decurrent tooth, tawny. Stipe 22–35 × 1.5–2.7 mm, central, cylindrical, equal in width from apex to base, surface brown, sometimes fading to white.

*Microscopic description*: Basidiospores [60/5/4], (6.4) 6.7–7.4 (7.8) × (3.9) 4.4–4.9 (5.4) µm, X = 7.0 ± 0.3 × 4.6 ± 0.3 µm, Q = 1.4–1.6, Qm = 1.5 ± 0.1, subrhomboid or ellipsoid in frontal view, oblong-ellipsoid in lateral view, thick-walled, smooth, with germ pore, yellowish brown in KOH. Basidia 19.5–23.3 × 6.4–7.5 µm, clavate to cylindrical, narrow at the base, thin-walled, hyaline, four-spored. Pleurocystidia type chrysocystidia, 33–40.3 × 13.4–17.7 µm, abundant, scattered, shape variable, often fusiform, clavate to broadly lageniform, with a short or long stalk base and a short neck, contents sometimes present in Congo red, thin-walled, hyaline. Cheilocystidia 14.7–19.9 × 5.3–7.1 µm, abundant, typically narrowly lageniform, subfusiform or subutriform, usually with a short neck, thin-walled, hyaline. No clamp connections.

*Habitat and Habit*: Saprotrophic, scattered on decaying wood and dead branches in mixed coniferous-broadleaved forest. Currently found only in the Shennongjia area of Hubei province, China.

*Additional specimens examined*: China, Hubei province, Shennongjia Forestry District, Shennong Peak Watchtower, 31°27′30″ N, 110°16′13″ E, 2886 m a.s.l., 26 August 2022, X.Y. Zhu, *ZRL20220900* (HMAS 282621); China, Hubei province, Shennongjia Forestry District, Shennong Peak Watchtower, 31°27′30″ N, 110°16′13″ E, 2886 m a.s.l., 20 June 2023, J.X. Li, *ZRL20230157* (HMAS 282622); China, Hubei province, Shennongjia Forestry District, Shennong Peak Watchtower, 31°27′30″ N, 110°16′13″ E, 2886 m a.s.l., 20 June 2023, J.X. Li, *ZRL20230186* (HMAS 282623).

*Notes*: Phylogenetic analyses place *D*. *lignicola* in a well-supported clade and close to *D*. *cokeriana* (A.H.Sm. and Hesler) (Ram.-Cruz and A. Cortés-Pérez), *D*. *thailandensis* (E. Horak, Guzmán, and Desjardin) (Ram.-Cruz and Guzmán), *D*. *overeemii* (E. Horak and Desjardin) (Desjardin and B.A. Perry), and *D*. *flava* (Y.Y. Shen and Y.B. Song) (Figure 2). However, *D*. *lignicola* can be clearly differentiated from each of these related taxa based on distinct macroscopic and microscopic features. *Deconica cokeriana* exhibits an inflexed to involute pileus margin and juvenile basidiomata displaying a completely flocculose surface or white fibrillose veil remnants on half of the pileus [58], whereas *D*. *lignicola* has a campanulate to plane-convex pileus with a straight margin and a smooth surface in its immature state. *Deconica thailandensis* has a smaller pileus, with a diameter of only 6–15 mm, which sets it apart from the other species in the clade. Although similar in structure to *D*. *lignicola*, *D*. *thailandensis* bears persistent white veil remnants on the pileus margin, with distinctive, persistent fibrils of the veil near the margin, rendering the margin appendiculate due to persistent fibrillose squamules [59]. *Deconica overeemii* shares a similar pileus shape to *D*. *lignicola*, but lacks true chrysocystidia, a fundamental microscopic difference. Furthermore, its pileus is not striate, unlike the typically striate pileus of *D*. *lignicola* [60]. *Deconica flava* can be separated by its floccose pileus surface, often with white fibrillose patches [26]. Both *D. flava* and *D. lignicola* possess chrysocystidia that are broadly lageniform with a mucro, but the chrysocystidia of *D. lignicola* (33–40.3 × 13.4–17.7 µm) are significantly larger than those of *D. flava* (15.2–26.8 × 5.6–13.0 µm) [26,59,60].

**Figure 5:**
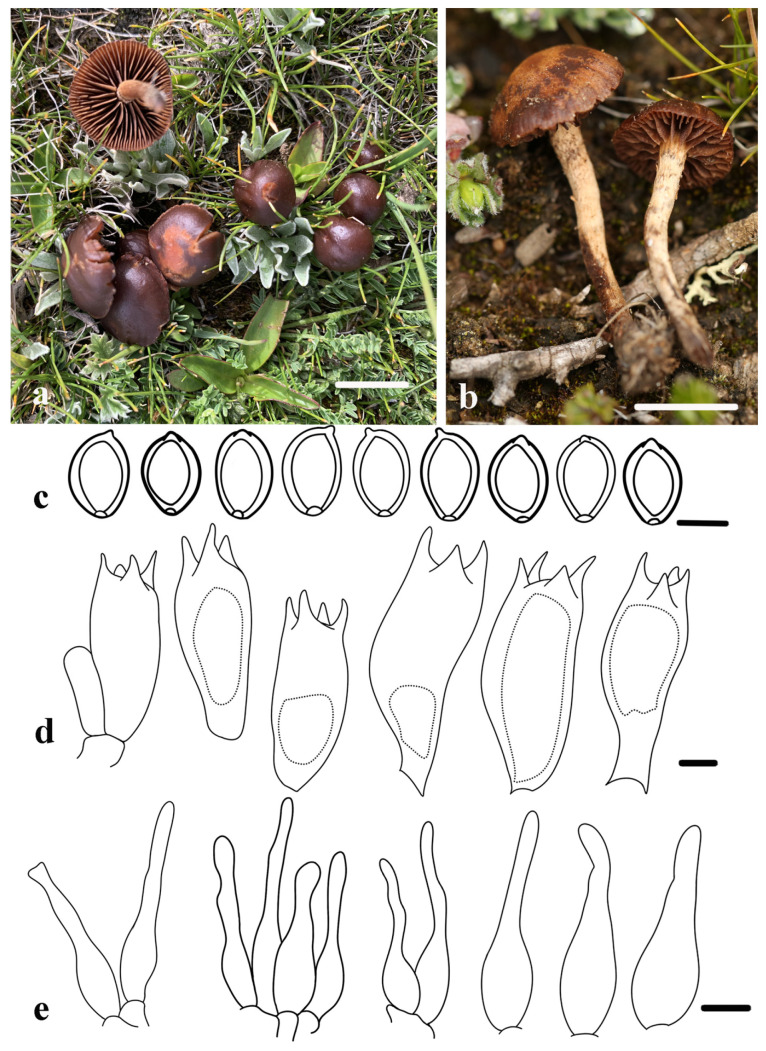
Morphology of *Deconica shannanensis*. (**a**) Basidiomata (holotype, HMAS 282624). (**b**) Basidiomata (HMAS 282625). (**c**) Basidiospores. (**d**) Basidia. (**e**) Cheilocystidia. Scale bars: (**a**,**b**) = 1 cm, (**c**–**e**) = 5 µm.

***Deconica shannanensis*** R.L. Zhao, H.Y. Feng, J.X. Li, and B. Cao, sp. nov. Figure 5.

Fungal Names: FN573017

*Etymology*: *shannanensis* (Latin) referring to Shannan City, Xizang, China, where the holotype was collected.

*Type*: China, Xizang, Shannan City, Yamdrok Yumtso, 29°11′38″ N, 90°37′19″ E, 4770 m a.s.l., 30 July 2021, M.Q. He, *ZRL20211203* (holotype HMAS 282624), Genbank accession: PV940843 (ITS), PV940893 (LSU), PX376973 (*rpb2*), PX401774 (*tef1-α*).

*Diagnosis*: *Deconica shannanensis* is characterized by its reddish-brown to dark brown, non-papillate basidiomata and brown lamellae with white squamulose veil remnants; Microscopically, it possesses ellipsoid or ovoid basidiospores with a distinct germ pore, abundant narrowly lageniform to lageniform cheilocystidia with a long neck, and no pleurocystidia.

*Macroscopic description*: Pileus 5–18 mm, initially hemispherical, expanding to convex at maturity, without papilla, dark brown or reddish-brown; smooth pileus of juvenile basidiomata, frequently developing areolate, ochraceous maculae at maturity; margin slightly inrolled, irregular. Context thin, light brown. Lamellae adnate, rugose, with lamellulae of unequal length situated inbetween, with large intervals, finely serrated edges, brown. Stipe 8–22 × 2.3–3.2 mm, central, cylindrical, equal in width, surface beige, rough, with white and brown fibrillose squamules at the apex.

*Microscopic description*: Basidiospores [60/3/2], (7.0) 7.6–8.8 (9.9) × (4.5) 5.0–6.0 (6.5) µm, X = 8.2 ± 0.6 × 5.5 ± 0.5 µm, Q = (1.3)1.4–1.6 (1.8), Qm = 1.5 ± 0.1, amygdaliform or ellipsoid in frontal view, ellipsoid to ovoid in lateral view, wall slightly thickened, smooth, with germ pore, color from dark yellow to yellowish-brown in KOH. Basidia 21.1–23.7 × 8.1–9.4 µm, cylindrical to clavate, thin-walled, hyaline, four-spored. Cheilocystidia abundant, 21.2–30.8 × 6.1–7.8 µm, narrowly lageniform to lageniform, typically with a long neck, thin-walled, hyaline. No pleurocystidia. No clamp connections.

*Habitat and Habit*: Saprotrophic, scattered or gregarious on cattle dung in alpine meadows. Currently found only in Xizang, China.

*Additional specimens examined*: China, Xizang, Rikaze area, Yadong County, Pari Town Plateau Meadow, 27°45′23″ N, 89°6′18″ E, 4330 m a.s.l., 28 July 2022, M.Q. He, *ZRL20220118* (HMAS 282625).

*Notes*: Phylogenetic analyses place *D*. *shannanensis* as the sister taxon to *D*. *inquilina* in Subclade A3, although this relationship lacks statistical support (Figure 2). Despite this phylogenetic proximity, the two species are distinctly separated by both morphological and ecological traits. Macroscopically, *D*. *shannanensis* exhibits a non-striate pileus that lacks a separable pellicle, contrasting with the translucent-striate pileus and separable pellicle of *D*. *inquilina*. Furthermore, *D*. *shannanensis* possesses a typically larger pileus (5–18 mm) but a shorter and thicker stipe, whereas *D*. *inquilina* has a smaller pileus (4–10.5 mm) and a more slender, elongated stipe (15–35 × 1–2 mm) [25]; From an ecological perspective, their ecological niches are distinctly different: *D*. *shannanensis* is a coprophilous species inhabiting high-altitude meadows, while *D*. *inquilina* grows on leaf litter and moss [6]. When compared to other members of Subclade A3, including *D*. *xeroderma*, *D*. *ovispora*, *D*. *subviscida*, and *D*. *castanella*, *D*. *shannanensis* remains morphologically distinguishable. Its hemispherical to convex brown pileus, which develops rough patches on the surface upon maturity, whereas *D*. *xeroderma* has a smooth pileus surface, often covered by a pale yellow, silky veil [61]. Additionally, while species such as *D*. *ovispora*, *D*. *subviscida*, and *D*. *castanella* may share a brown pileus coloration, they consistently exhibit distinct striations and a central, low convexity. Furthermore, the pileus margin of both *D*. *subviscida* and *D*. *castanella* is frequently adorned with an appendiculate veil, and *D*. *castanella* is characterized by a dense covering of small white patches on the pileus surface [18,19,62,63]. These consistent morphological differences effectively delineate *D*. *shannanensis* from its phylogenetic relatives within the subclade.

## 4. Discussion

Despite significant advances in the study of *Psilocybe* and *Deconica*, much of their diversity remains undiscovered. Current taxonomic efforts have formally described only a fraction of the species within these genera, with many more awaiting discovery and description. This is especially true for *Deconica*, although early estimates suggested the genus could comprise over 100 species, the number of currently recognized species is far lower [64,65]. Similarly, the species diversity, distribution, and phylogenetic relationships of both genera within China remain largely unexplored. For instance, recent editions of the *Flora Fungorum Sinicorum* list only a handful of *Psilocybe* species, and while several new taxa have been recently described (*P*. *zhushanensis*, *P*. *ningshanensis*), the total count remains limited [23,24,48]. The situation is even more critical for *Deconica*, with only five species reported from China to date. This significant gap between regional records and global diversity estimates, compounded by a lack of comprehensive molecular data, underscores the urgent need for further taxonomic investigation in the region, which this study begins to address.

In this study, based on an integrative taxonomic examination of 64 specimens, we identified nine known species across the two genera under investigation, as well as three new species to science. The known species include *P*. *cubensis*, *P*. *cyanofibrillosa*, *P*. *keralensis*, *P*. *ningshanensis*, *P*. *ruiliensis*, *P*. *subcaerulipes*, *D*. *austrosinensis*, *D*. *coprophila*, and *D*. *xeroderma*. Additionally, several collections were provisionally identified as *Psilocybe* sp. and *Deconica* sp., which may represent new taxonomic entities, though these findings require further investigation. Notably, their morphoanatomic features and phylogenetic affinities to known species were not discussed in detail. The discovery of *P*. *striata* in Chongqing is particularly noteworthy, as it represents a new locality record for *Psilocybe* in China, expanding its known distribution beyond previously documented areas like Yunnan and Xizang [27,28,48]. Meanwhile, the discoveries of *D*. *lignicola* and *D*. *shannanensis* enrich the known diversity of their respective lineages within China. Notably, *D. shannanensis* is one of the *Deconica* species recorded in high-altitude meadows, further contributing to our understanding of the genus’ ecological diversity. These novel taxa raise critical questions about the historical biogeography and ecological plasticity of these fungi, suggesting their adaptive capacities may be broader than previously recognized.

The concept of *Psilocybe* s.str., as defined by Redhead et al. (2007), restricts the genus to species that exhibit a bluing reaction upon mechanical damage, a trait associated with psychoactive compounds [2]. Phylogenetically, *P*. *striata* is robustly placed within *Psilocybe* Clade D, a group where all confirmed members are known to be psychoactive [6,8,14]. Given its phylogenetic placement within this clade and its strong bluing reaction—a widely recognized macroscopic indicator for psilocybin—we hypothesize that *P*. *striata* may also produce these compounds and could therefore be considered potentially toxic. This morphological and phylogenetic evidence reinforces the utility of the bluing reaction as a reliable macroscopic indicator of potential toxicity within *Psilocybe*, particularly when interpreted in a phylogenetic context. Within *Deconica*, Subclade A1 (subclade “chrysocystidiatae”) is primarily defined by the presence of chrysocystidia, specialized cystidia with golden-yellow contents. However, the absence of chrysocystidia in some phylogenetically nested species, such as *D*. *fuscobrunnea*, *D*. *furfuracea,* and *D*. *austrosinensis*, demonstrates that the presence of chrysocystidia is not a stable diagnostic character for this entire lineage and should not be solely relied upon for classification [6,18].

In conclusion, this study advances the taxonomy of *Psilocybe* and *Deconica* in China by describing three new species and confirming nine known species through integrated morphological and phylogenetic analyses. Our work reveals the rich, yet largely undiscovered, diversity of these genera in the montane and alpine ecosystems of Chongqing and Xizang, emphasizing the need for continued exploration. The findings provide a crucial foundation for future research into the ecology, biogeography, and evolution of these fungi.

## Data Availability

All sequence data are available in NCBI GenBank following the accession numbers in the Supplementary Materials.

## Supplementary Materials

The following supporting information can be downloaded at: https://zenodo.org/records/17667185, Figure S1: Maximum likelihood (ML) tree of *Psilocybe* inferred from ITS, nrLSU, *rpb2* and *tef1-α* sequences; Figure S2: Bayesian Inference (BI) tree of *Deconica* inferred from ITS, nrLSU, *rpb2* and *tef1-α* sequences; Table S1: Taxa, vouchers and GenBank accession numbers of *Psilocybe* taxa used in this study; Table S2: Taxa, vouchers and GenBank accession numbers of *Deconica* taxa used in this study. Additionally, the phylogenetic trees and sequence alignments have been deposited in the National Microbiology Data Center, NMDC. The official number is NMDCX0002180.
